# Inhibition of the Combinatorial Signaling of Transforming Growth Factor-Beta and NOTCH Promotes Myotube Formation of Human Pluripotent Stem Cell-Derived Skeletal Muscle Progenitor Cells

**DOI:** 10.3390/cells10071649

**Published:** 2021-06-30

**Authors:** In Young Choi, Ho Tae Lim, Young Hyun Che, Gabsang Lee, Yong Jun Kim

**Affiliations:** 1 Department of Medicine, Graduate School, Kyung Hee University, Seoul 02447, Korea; dls202@khu.ac.kr; 2 The Institute for Cell Engineering, School of Medicine, Johns Hopkins University, Baltimore, MD 21205, USA; hlim29@jhmi.edu; 3 Department of Biomedical Science, Graduate School, Kyung Hee University, Seoul 02447, Korea; mojagom@hanmail.net; 4 Department of Neurology, School of Medicine, Johns Hopkins University, Baltimore, MD 21205, USA; 5 The Solomon H. Snyder Department of Neuroscience, School of Medicine, Johns Hopkins University, Baltimore, MD 21205, USA; 6 Department of Pathology, College of Medicine, Kyung Hee University, Seoul 02447, Korea

**Keywords:** human pluripotent stem cell-derived skeletal muscle, in vitro drug screening platform, Duchenne muscular dystrophy

## Abstract

Understanding the signaling pathways that regulate the final differentiation of human myoblasts is essential for successful cell transplantation and drug screening for the treatment of muscular dystrophy. In an effort to improve myotube formation from hiPSC-derived myoblasts, we validated a collection of 13 small molecules in a newly established in vitro screening platform for the assessment of myotube formation. The analysis of myotube formation as measured by the fusion index showed that the combinational inhibition of the TGFβ signaling with NOTCH signaling enhances the ability of multi-nucleated myotube production. Combinational treatment of inhibitors for TGFβ and NOTCH signaling pathways improved myotube formation in a dose-dependent manner. This effect was achieved by inhibiting the combinatorial mechanism of signaling. The combination treatment of small molecules effective in inducing multinucleated myotubes was validated in healthy human primary myoblasts. In addition, it was also applied to DMD patient iPSC-derived myoblasts to enhance the generation of multinucleated myotubes.

## 1. Introduction

Skeletal muscle is one of the tissues in the body that physiologically repeats damage and regeneration in response to external stimuli. Rapid regeneration of damaged muscle cells is essential for restoring physiological abilities involved in various body controls such as exercise, breathing, and thermoregulation from the injured conditions [1]. For the consistent regeneration of injured tissues, the human body operates a tissue regeneration system called tissue stem cells, and a number of studies have suggested that the skeletal muscle progenitor cells (SMPCs) role as a regenerating population in the muscle tissue [2]. Although researches based on primary adult stem cells have been attempted to investigate the association between SMPCs and muscle development [3,4] or muscle regeneration [5,6,7,8], studies to understand the cellular and molecular behaviors of human SMPCs have not yet been conducted enough due to limitations in the size of the stem cell population and in the lack of knowledge to maintain the fate of stem cells in vitro.

Our previous work using human pluripotent stem cells (hPSCs) has allowed the understanding of pathophysiological mechanisms of human skeletal muscle development and related diseases, as well as validating the effects on targeting or transplanting SMPCs as therapeutics for skeletal muscle degenerating diseases, Duchenne muscular dystrophy (DMD) [9]. While attempts to employ SMPCs have made the treatment of muscle disorders feasible, further research into how to promote muscle production from SMPCs is an advantageous practical approach. Given the results of recent research on the pathophysiology of muscle tissue, targeting the transforming growth factor-beta (TGF-β) or NOTCH signaling pathway, which is responsible for atrophy or hypertrophy of skeletal muscle tissue, might be possible therapeutic targets for muscle reconstruction. However, it is essentially required to dissect developmental stages in vitro to validate effective cellular stages that are sensitive to molecular signal regulation.

In this study, we screened small molecule compounds based on the imaging-based evaluation system for myotube formation to derive effective target molecules which regulate signaling pathways, including TGF-β and NOTCH to enhance the myotube formation of SMPCs. Inhibition of each or combinatorial molecules escalated the formation of multinucleated myotubes from both the human induced pluripotent stem cell (hiPSC)-derived SMPCs and human primary myoblasts, as well as DMD patients hiPSC-derived SMPCs. Our results indicate that the primary cellular target of TGF-β or NOTCH, which promotes changes in muscle mass under pathophysiological conditions, is SMPCs rather than mature muscle cells, and further suggest both signaling molecules as candidate targets to stimulate resident or transplanted SMPCs in the patient’s tissue to encourage muscle production.

## 2. Materials and Methods

### 2.1. Cell Culture of hiPSCs

The healthy hiPSC (GM00024) and DMD patient’s iPSC (GM05169) were established and cultured using previously published methods [9,10]. Briefly, hiPSCs were maintained on mitomycin-C treated MEFs (Life Technologies, Carlsbad, CA, USA) at 12,000 to 15,000 cells/cm^2^ in a gelatin-coated cell culture dish, and the medium was changed every day with weekly passaging using either 6 U/mL Dispase (Gibco, Hongkong, China) or mechanical detachment. The medium for maintaining hiPSCs contained DMEM/F12, 20% knockout serum replacement, 1 mM L glutamine, 100 µM MEM non-essential amino acids, 10 ng/mL of FGF2, and 0.1 mM β-mercaptoethanol (Life Technologies, Carlsbad, CA, USA). Cells were monitored free of contamination every other week using a MycoAlert^TM^ Mycoplasma Detection Kit (Lonza, Basel, Switzerland).

### 2.2. In Vitro Skeletal Muscle Differentiation

Skeletal muscle specification was performed as previously described [9,11]. To initiate myogenic differentiation, hiPSCs were dissociated to single cells using Accutase (Sigma Aldrich, St. Louis, MO, USA) for 20 min incubation, and placed on a gelatin-coated dish for 30 min to remove MEFs. Non-adherent cells were collected, counted, and plated on a 1% Geltrex (Thermo Fisher, Waltham, MA, USA)-coated dish, at a density of 1.5 × 10^5^ cells per well of a 24-well plate, in the presence of filtered MEF-conditioned N2 medium containing 10 ng/mL of FGF2, and 10 μM of Y-27632 (Cayman Chemical, Ann Arbor, MI, USA). At 70% of confluence, N2 medium with CHIR99021 (3 μM) was added and changed every other day. On day 4, N2 medium with DAPT (10 μM) treatment was added until day 12. After that, the cells were fed with an N2 medium.

### 2.3. Isolation of NCAM+/HNK- Myoblasts from hiPSCs Derived Differentiated Cells

After 25 to 30 days of skeletal muscle specification, the differentiated cells were purified with the FACS cell sorting strategy. This purification process was performed as previously described [9]. In brief, cells were dissociated with the enzyme and stained with primary antibodies (NCAM, Santacruz; HNK1, Sigma Aldrich, St. Louis, MO, USA) in 15 min. After the washing step, secondary antibodies were added for another 15 min. Finally, the stained cells were transferred to the FACS tube through the cell strainer. The sorting was done with FACSAria System (BD Biosciences, San Jose, CA, USA) to obtain NCAM+/HNK- fraction.

### 2.4. Cell Culture of Myoblasts and Myotube Formation

The NCAM+/HNK1- myoblasts were maintained in a humidified incubator containing 5% CO_2_ at 37 °C. The myoblasts were grown in N2 medium supplemented with 5% fetal bovine serum (FBS), 10 ng/mL FGF-2, and 100 ng/mL FGF-8. The medium was changed every other day. For passaging every week, cells were dissociated with trypsin for 5 min, washed once with N2 medium. The human primary myoblast (03Ubic) was kindly provided by Senator Paul D. Wellstone Muscular Dystrophy Cooperative Research Center. Cells expressing CD56 were isolated from a 42-year-old healthy female donor obtained from the 15 cohorts studied in a previous study [12] under the regulation of Johns Hopkins School of Medicine Institutional Review Board. The culture primary myoblasts, To culture primary myoblasts, we adopted the protocol from Emerson’s laboratory [13]. Cells were grown in HMP growth medium containing 20% FBS, 0.5% Chick Embryo Extract, 1% antibiotics/antimycotics, and 1.2 mM CaCl2 in Ham’s F10 medium. To induce myotube formation, primary myoblasts were cultured with a differentiation medium containing 2% horse serum, 2mM L-glutamine, 20mM HEPES, and 1% antibiotics/antimycotics in Ham’s F10 medium. To induce myotube formation, expanded NCAM+/HNK1- myoblasts were plated in the same cell number with serum-free N2 medium. At this point, small molecules were added in the fusion-induction medium to experiments. After 10 days with serum-free N2 medium, cells were fixed and stained with myotube-specific markers and were imaged by using Nikon Eclipse TE2000-E fluorescence microscopy.

### 2.5. Small Molecule Screening for Enhancing Myotube Formation

The cultured NCAM+/HNK1- myoblasts were seeded onto 48-well plates and incubated in presence of an expansion medium. At 100% confluency, the culture medium was switched to a differentiation medium, either supplemented or not with small molecules, in a different final concentration (Table 1). For the myotube formation, the medium was changed every other day. After ten days later, cells were stained for myotube markers or harvested for qRT-PCR analysis.

### 2.6. Growth Curve Assay

The cultured cells were dissociated from the flask by trypsin (Sigma Aldrich) to digest and after wash with N2 medium, seeded into 24-well cell culture plates (1 × 10^4^ cells/well). The medium change was done every other day with N2 medium supplemented with 5% fetal bovine serum (FBS), 10 ng/mL FGF-2, and 100 ng/mL FGF-8. Plated cells were dissociated from wells with 0.25% trypsin and counted each day using a hemocytometer. Cell growth curves were drawn from live-cell numbers for seven days.

### 2.7. Cell Proliferation and Apoptosis Assay

Proliferation and apoptosis were determined by staining using Annexin V:APC or Ki-67 (Invitrogen, Waltham, MA, USA). Briefly, myoblasts were seeded into 48-well cell culture plates (5 × 10^4^ cells/well) for 24 h. For apoptosis assay, cells were dissociated from wells with 0.25% trypsin, spun at 1200 rpm for 5 min. Then the cells were resuspended in FACS buffer with 1 µL Annexin V::APC antibody for 15 min. Cells were analyzed using the FACS Calibur System (BD Biosciences, San Jose, CA, USA). The relative proportion of Annexin V-positive cells, representing apoptotic cells, was determined using Flow Jo software (Tree Star Inc., OR, USA). For proliferation assay, the plated cells were fixed and stained with a Ki-67 antibody. The percentage was calculated as the ratio of the number of Ki-67 positive cells over the number of total nuclei in the image.

### 2.8. Immunofluorescence Analysis

For immunofluorescence, live cells were fixed with 4% paraformaldehyde for 15 min and washed with PBS. Then permeabilized with 0.1% Triton X-100 in 0.5% BSA included PBS solution. Primary antibodies (NANOG, Cell signaling; PAX7, DSHB; MF20, DSHB; DESMIN, DAKO) were applied after blocking with 0.5% BSA solution and incubated overnight at 4 °C. After three times washing with PBS, appropriate secondary antibodies were applied for 2 h at RT. Finally, nuclei were stained with DAPI for 15 min. For imaging, the stained cells were visualized with a microscope (Nikon TE2000).

### 2.9. Fusion Index and Ratio of Area Analysis

Fusion index was measured and analyzed as previously described [9]. Briefly, to determine fusion index, cells were induced final differentiation for 10 days with N2 medium with/without small molecules and stained with MF20 antibody. Fusion index was calculated as the ratio of the number of nuclei inside MF20-positive myotubes over the number of total nuclei in the image. To normalize for differences between plates or experimental batches, all trials included untreated negative control wells and positive wells treated with fusion induction medium only. The results of the fusion index of the experimental group were accepted when the fusion index of the negative control well was 1 or less and the fusion index of the positive control well was 10 or more. The ratio of the area of MF20/DAPI staining was quantified using Image J.

### 2.10. Quantitative Real-Time PCR Analysis

The qRT-PCR experiments were performed as previously described [9]. Briefly, total RNA was extracted from harvested cell pellets using TRIzol Reagent (Life Technologies), and 1 µg of total RNA was reverse transcribed using the High Capacity cDNA Reverse Transcription Kit (Applied Biosystems, Waltham, MA, USA). The qRT-PCR mixtures were prepared with SYBR Green PCR Master Mix (Kapa Biosystem), and the reactions were performed using the MastercyclerⓇ ep Realplex^2^ (Eppendorf) with indicated primer set (*PAX7* f: AGGCCTTTGAGAGGACCCAC, r: CTGAACCAGACCTGCACACG; MYF5 f: GCCTGAAGAAGGTCAACCAG, r: AGGTTGCTCTGAGGAGGTGA; *MYOG* f: GGTGCCCAGCGAATG, r: TGATGCTGTCCACGATCGA; *TTN* f: TCAAGACAAACCCGAATTGA, r: GAGGCACTTCAGGACCTGTG; *MYH2* f: CCTGCTGTGCTGTACAACCT, r: GGTTGGCACTGATGATTTGA; *MEF3C* f: TGGATGAACGTAACAGACAGG, r: ACTGTTGTGGCTGGACACTG; *HES1* f: AAGAAAGATAGCTCGCGGCA, r: TACTTCCCCAGCACACTTGG; *HES5* f: TCCTGGAGATGGCTGTCAGCTA, r: CGTGGAGCGTCAGGAACTGCA, *HEY1* f: CCTTCCCCTTCTCTTTCGGC, r: AAAAGCTCCGATCTCCGTCC, *HEYL* f: AGACCGCATCAACAGTAGCC, r: GTGATCCACCGTCATCTGC, *JAG1* f: TGCTACAACCGTGCCAGTGACT, r: TCAGGTGTGTCGTTGGAAGCCA). The transcription levels were assessed by normalizing to *GAPDH* expression (*GAPDH* f: CGAGATCCCTCCAAAATCAA, r: TTCTAGACGGCAGGTCAGGT).

### 2.11. Statistical Analysis

All statistical analyses in this study were performed by Prism 6 (GraphPad, San Diego, CA, USA). The values were the results of at least three independent experiments, with multiple replicates of each, and reported as the mean ± SEM. Differences between the two samples were analyzed for significance using the unpaired t-test.

## 3. Results

### 3.1. Establishment of the Screening Platform Based on hPSC-Derived Skeletal Muscle Differentiation

**Figure 1:**
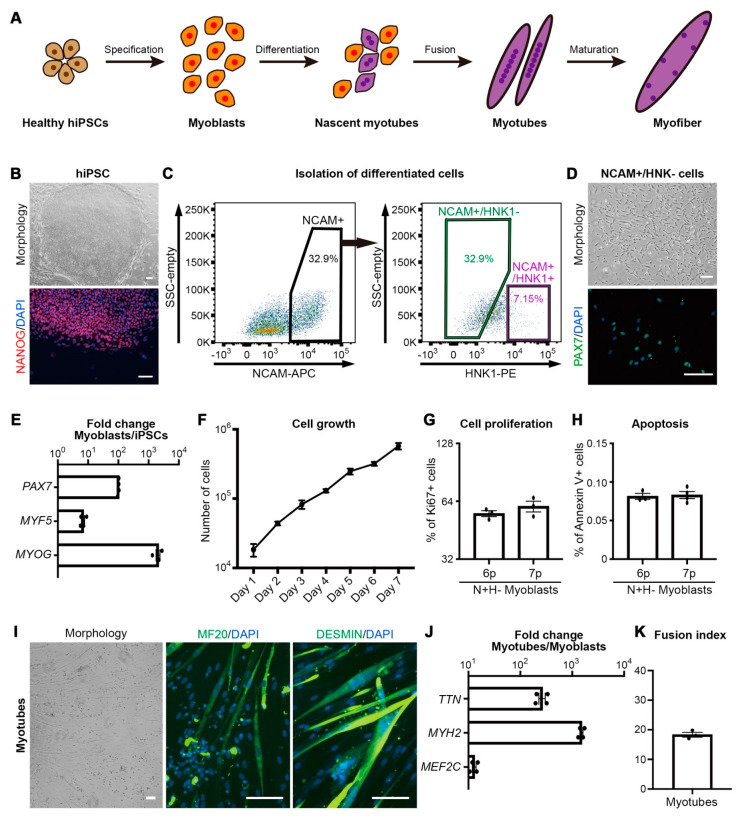
In vitro screening platform for myotube formation using human pluripotent stem cell-derived myoblasts. (**A**) The schematic illustration of the embryonic myogenesis of hiPSCs. (**B**) Representative colony morphology of undifferentiated healthy hiPSCs and expression of pluripotency marker NANOG. (**C**) Representative FACS plots for NCAM+/HNK1- cell population. (**D**) Representative image of NCAM+/HNK1- (N+H-) myoblasts and immunohistochemistry of *PAX7* in N+H- myoblasts. (**E**) Fold changes of expression levels with myogenic-specific marker genes, *PAX7*, *MYH5*, and *MYOG* in the myoblasts/undifferentiated hiPSCs. (**F**) The cell proliferation rate of N+H- myoblasts after sorting. (**G**) The percentage of Ki67+ cells in the N+H- population. (**H**) The percentage of Annexin V+ cells in the N+H- population. (**I**) Representative image of myotube from expanded myoblasts (hiPSC-derived). And myotube formation ability of N+H- cells confirmed by MF20 and DESMIN expression. (**J**) Myotube-related gene expression levels in the myotube/myoblasts. (**K**) The fusion index of the myotube which calculated as a ratio of the number of nuclei inside myotubes to the number of total nuclei ×100 after myotube formation. Scale bars = 100 um.

To establish the screening platform for the discovery of small molecules that promote human skeletal muscle development, we built a differentiation model that goes through the developmental processes from hiPSCs to myofibers (Figure 1a). Healthy hiPSCs maintaining a typical colony morphology of hPSCs along with the expression of pluripotent marker NANOG were differentiated into skeletal muscle cells (Figure 1b). Since the regeneration of damaged muscle is dependent on the abilities of myoblasts, such as proliferation and differentiation [14], we employed differentiated myoblasts to precisely distinguish the physiological target of small molecules on the abilities of myoblasts. After 30 days of differentiation, cells that express NCAM without HNK1 expression (N+H-) were isolated utilizing surface markers (Figure 1c) to exclude possible contamination of neural crest stem cells [15]. The purified N+H- cell population was confirmed as myoblasts with an expression of PAX7, a marker of developing myoblasts or adult muscle stem cells that contribute to myofiber regeneration (Figure 1d) [16], as well as enrichment of key marker genes for myoblasts such as *PAX7*, *MYF5*, and *MYOG* compared to undifferentiated hiPSCs (Figure 1e. Cultured myoblasts were successfully expanded after purification (Figure 1f). Expanded myoblasts sustained the ratio of Ki67-expressing cells more than 60% (Figure 1g) without significant apoptosis (Figure 1h) even after the seventh passage. To examine that hiPSC-derived myoblasts retain the capability of myotube formation, expanded myoblasts were further differentiated into myotubes followed by validation of efficiency for myotube formation. After 10 days of culture with serum depletion, elongated spindle-shaped and branching morphology containing three or more nuclei within a single membrane structure [17] accompanying expression of skeletal muscle cell markers such as MF20 and DESMIN was distinguished (Figure 1I). These myotubes showed significant enrichment of marker genes such as *TTN*, *MYH2,* and *MEF2C* compared to myoblasts before myotube formation (Figure 1J). The fusion index was calculated as the percentage of nuclei within the myotube relative to the total number of nuclei. The fusion index of differentiated myotubes from healthy hiPSC-derived myoblasts was 18.31 ± 0.818 which is consistent with the previous report of differentiated myotubes from human embryonic stem cells (Figure 1k) [9]. These data indicate that the hiPSC-derived N+H-cell population is a tissue stem cell possessing proliferation and differentiation capacity for myotube formation, and is suitable as a cellular resource for a small molecule screening platform to enhance muscle regeneration.

### 3.2. Small-Scale Screening for Small Molecules Enhancing In Vitro Myotube Formation

**Figure 2:**
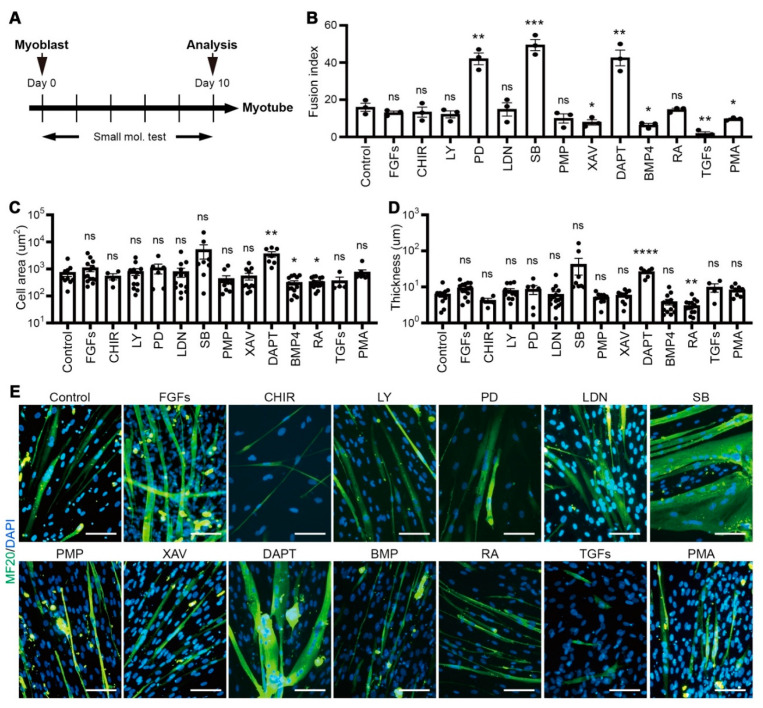

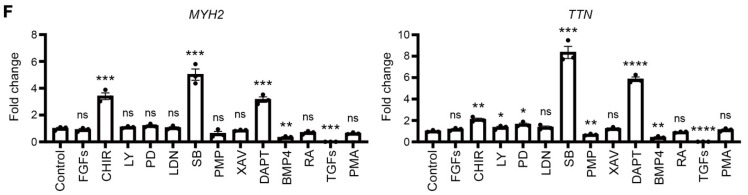
Small scale screening of small molecules for enhancing myoblasts fusion efficiency. (**A**) Experimental strategy for small molecule compound screening to enhance myotube formation. hiPSCs derived myoblasts were plated onto 48 well plates and cultured for 10 days with or without small molecules, and the fusion index was determined at day 10. (**B**) The fusion index analysis of myotubes that were differentiated with each small molecule. (**C**) The area of individual myotubes stained with MF20 with each small molecule. (**D**) The thickness of individual myotubes stained with MF20 with each small molecule. (**E**) Expression of MF20 on differentiated myotube with each small molecule treatment individually. (**F**) Fold changes of expression levels with myotube-specific marker genes, *MYH2* and *TTN* in 14 different conditions. Scale bars = 100 um. * *p* < 0.05, ** *p* < 0.01, *** *p* < 0.001, **** *p* < 0.0001, ns = non significance, One-way ANOVA.

To validate the role of key signaling pathways in the muscle generation process, we performed a small-scale screening of 13 small molecules regulating FGF (fibroblast growth factor), WNT (Wingless and Int), TGFβ (transforming growth factor factor-beta), BMP (bone morphogenetic protein), or NOTCH utilized in previous studies on myogenesis (Figure 2a) [9,18,19,20]. Medium containing each small molecule at different concentrations (Table 1) was changed every other day. After ten days of differentiation, myotubes generated by the addition of each small molecule were scored with a fusion index indicating the ability to form myotubes. Among the signaling regulator molecules, it was confirmed that the fusion index significantly increased in groups treated with FGF inhibitor PD-173074, TGFβ inhibitor SB-431542, or NOTCH inhibitor DAPT, respectively. Conversely, treatment of XAV-939, BMP4, TGFβ, or PMA (Phorbol 12-myristate 13-acetate) abolished the myotube formation compare to the non-treated control group (Figure 2b). In particular, myotubes generated in the presence of SB-431542 or DAPT were considerably thicker (Figure 2c) and denser (Figure 2d) than those of other groups although myotubes in all groups expressed MF20 (Figure 2e). We further profiled the mRNA expression levels of *MYH2* and *TTN* to endorse created myotubes. Interestingly, SB-431542 or DAPT induced myotubes showed elevated expression of both genes, suggesting efficient myotube formation (Figure 2f). Even though PD-173074 or CHIR-99021 encouraged the fusion index of myoblasts or the expression of *MYH2*, both conditions were excluded from appropriate conditions as both treatments were accompanied by significant apoptotic cell death (data not shown). Our data identified small molecules, TGFβ inhibitor, and NOTCH inhibitor, that stimulate myotube formation in terms of both quantity and quality.

### 3.3. Improved Efficiency of Myotube Formation by Combinational Treatment

**Figure 3:**
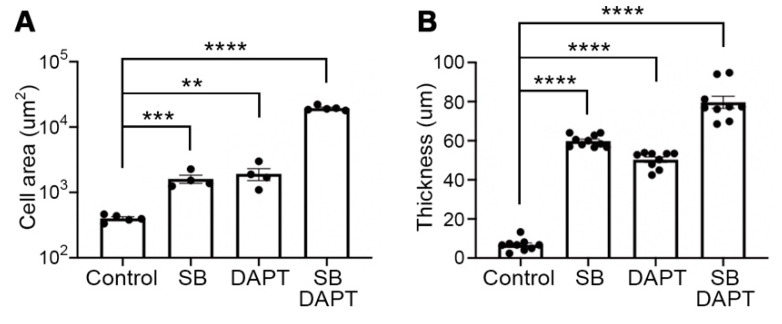

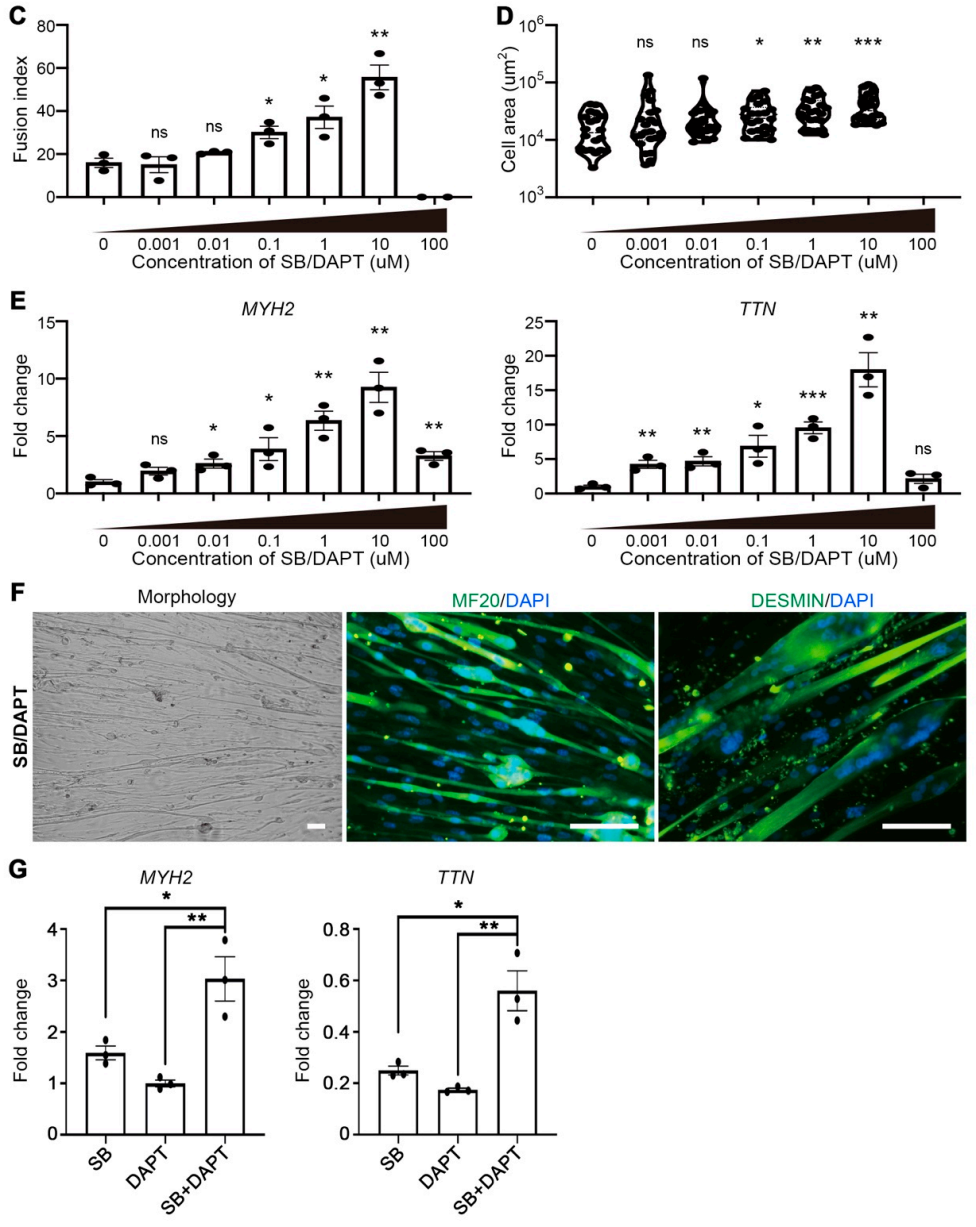
Augmentation of myotube formation and fusion ability by combinatorial treatment of SB-431542 and DAPT. (**A**) Quantification of cell area of myotubes following single or combinational treatment of SB-431542 and DAPT. (**B**) Quantification of the thickness of myotubes with SB-431542, DAPT, or combinational treatment. (**C**) Quantification of myotube for fusion index in dose-dependent combinational treatment of two small molecules. (**D**) The area of individual myotubes stained with MF20 in dose-dependent combinational treatment of two small molecules. (**E**) Fold changes of expression levels with myotube-specific marker genes, *MYH2* and *TTN* in dose-dependent combinational treatment of two small molecules. (**F**) Representative image of myotube differentiated with SB-431542 and DAPT (10 μM each) and immunofluorescence staining image of stained myotube with an antibody against MF20 and DESMIN with SB-431542 and DAPT (10 μM each). (**G**) Relative mRNA expression levels of myotube marker genes in single SB treatment, single DAPT treatment, and combinational SB+DAPT treatment. Scale bars = 100 um. * *p* < 0.05, ** *p* < 0.01, *** *p* < 0.001, **** *p* < 0.0001, ns = non significance, One-way ANOVA.

Since it is known that TGFβ and NOTCH cooperatively regulate various cellular mechanisms [21,22], we applied both signaling controls to assess the improvement of myotube formation. The myotube formation was induced for 10 days with single or combinational treatment of SB-431542 and DAPT. Interestingly, myotubes with combinational treatment showed an increased level of cell area (Figure 3a) and thickness (Figure 3b) compare to one in single treatment myotubes. Next, myotube formation was induced by different concentrations of SB-431542 and DAPT ranging from 0.001 uM to 100 uM. As expected, the fusion index exhibited a dose-dependent quantitative increase of myotube formation, un-til up to 10 uM of both molecules excluding clearance by apoptosis at concentrations of 100 uM (Figure 3c). In addition, the area of the multinucleated myotube at each concentration revealed a qualitative improvement of the produced myotubes due to both signaling regulators in a dose-dependent manner (Figure 3d). Furthermore, the escalation in mRNA expression of *MYH2* and *TTN* was detected in myotubes treated with 10 uM concentrations of both molecules (Figure 3e), which supports the results of quantitative and qualitative improvement of myotube formation as the concentration of signaling molecules was increased. The myotubes produced by the combinational treatment of two small molecules showed the expression of proper skeletal muscle markers, MF20 and DESMIN, as well as morphological properties of the myotube (Figure 3f). In addition, expression of myogenic marker genes, *MYH2* and *TTN*, was increased in combinational treatment-induced myotubes compare to those in every single molecule treated myotubes (Figure 3g).

### 3.4. Physiological Role of TGFβ and NOTCH during Myogenesis

**Figure 4:**
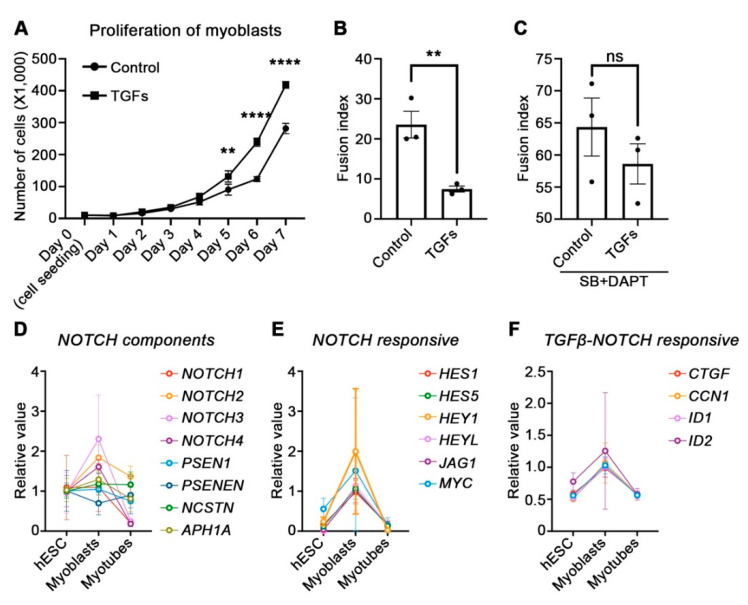

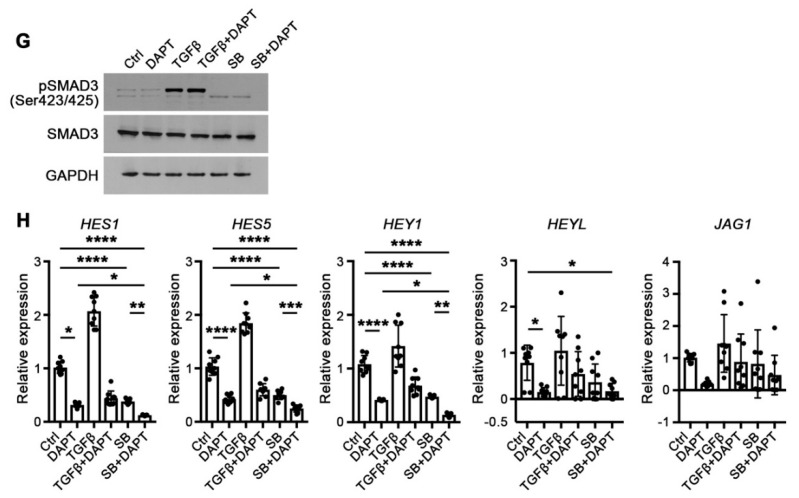
physiological role of TGFβ and NOTCH in the myogenic process. (**A**) The proliferation of hPSC-derived myoblasts with or without TGFs (TGFβ1, TGFβ2, and TGFβ3). (**B**) The fusion index was calculated after treatment of TGFs for 7 days. (**C**) The fusion index of control or TGFs-treated myoblasts with SB-431542 and DAPT. (**D**) Gene expression level of NOTCH component factors was analyzed using an RNA sequencing database for myogenesis (www.myogenesis.net). (**E**) Gene expression level of downstream factors of NOTCH was analyzed using RNA sequencing database for myogenesis (www.myogenesis.net). (**F**) The expression level of genes under the co-regulation of TGFβ and NOTCH was analyzed using an RNA sequencing database for myogenesis (www.myogenesis.net). (**G**) Immunoblotting for confirmation of SMAD3 activity by single or combinational treatment of DAPT with TGFβ or SB-431542. (**H**) The transcription level of NOTCH-responsive genes by single or combinational treatment of DAPT with TGFβ or SB-431542. * *p* < 0.05, ** *p* < 0.01, *** *p* < 0.001, **** *p* < 0.0001, ns = non significance, Unpaired *t-test* or One-way ANOVA.

A number of studies using animal models have reported that TGFβ promotes fibrosis by activating mesenchymal cells, resulting in impaired regeneration of damaged muscle tissues [23]. As our data showed that TGFβ inhibited myotube formation without mesenchymal cells in Figure 2b, we investigated the biological role of TGFβ in the stage of the myoblast rather than myotube formation during muscle development. Myoblasts were incubated with TGFβ prior to the myotube formation, and intriguingly, TGFβ encouraged the proliferation of myoblasts to compare to the non-treated one (Figure 4a). Although expanded myoblasts by adding TGFβ showed decreased fusion index (Figure 4b), TGFβ treated myoblasts obtained improved fusion ability by treatment with SB-431549 and DAPT (Figure 4c). To support the results of promoting myotube formation by NOTCH inhibition, we profiled the expression of NOTCH-related genes, which have been reported for regulation by TGFβ [24], in a myogenic transcriptional landscape established from our previous (www.myogenesis.net, accessed on 10 May 2021) [11]. Indeed, informatics data revealed that NOTCH genes and gamma-secretase component genes involved in the NOTCH signaling pathway, such as *NOTCH1*, *NOTCH2*, *NOTCH3*, *NOTCH4*, *PSEN1*, and *APH1A* were significantly reduced in myotubes than in myoblasts (Figure 4d). Additionally, a decrease of downstream genes of the NOTCH signaling pathway such as *HES1*, *HES5*, *HEY1*, *HEYL*, *JAG1*, and *MYC* was discovered (Figure 4e). Remarkably, among the target genes of TGFβ, it was confirmed that genes such as *CTGF*, *CCN1*, *ID1*, and *ID2*, which are positively regulated by crosstalk with NOTCH, were also declined along with myotubes formation from myoblasts (Figure 4f). To determine how inhibition of TGFβ and NOTCH promotes myotube formation, the transcription level of NOTCH-responsive genes by DAPT treatment along with activation or inhibition of TGFβ was validated. The addition of TGFβ or SB-431542 regulated SMAD3 activity in hiPSC-derived myoblasts [24], and cells were treated with or without DAPT (Figure 4g). Interestingly, treatment with SB-431542 alone downregulated the transcription levels of NOTCH-responsive genes such as *HES1*, *HES5*, and *HEY1*, comparable to those in the group that inhibited NOTCH by adding DAPT alone. Furthermore, the combinational treatment of SB-431542 and DAPT diminished the expression of the NOTCH-responsive genes more effectively compared to the results of DAPT or SB single treatment (Figure 4h). Taken together, these data provide evidence that TGFβ promotes proliferation of myoblasts, and that inhibiting TGFβ cooperate with the NOTCH pathway to enhance myotube formation.

### 3.5. Application of Small Molecules Enhances Myotube Formation of Primary Myoblasts and Patient’s Myoblasts

**Figure 5:**
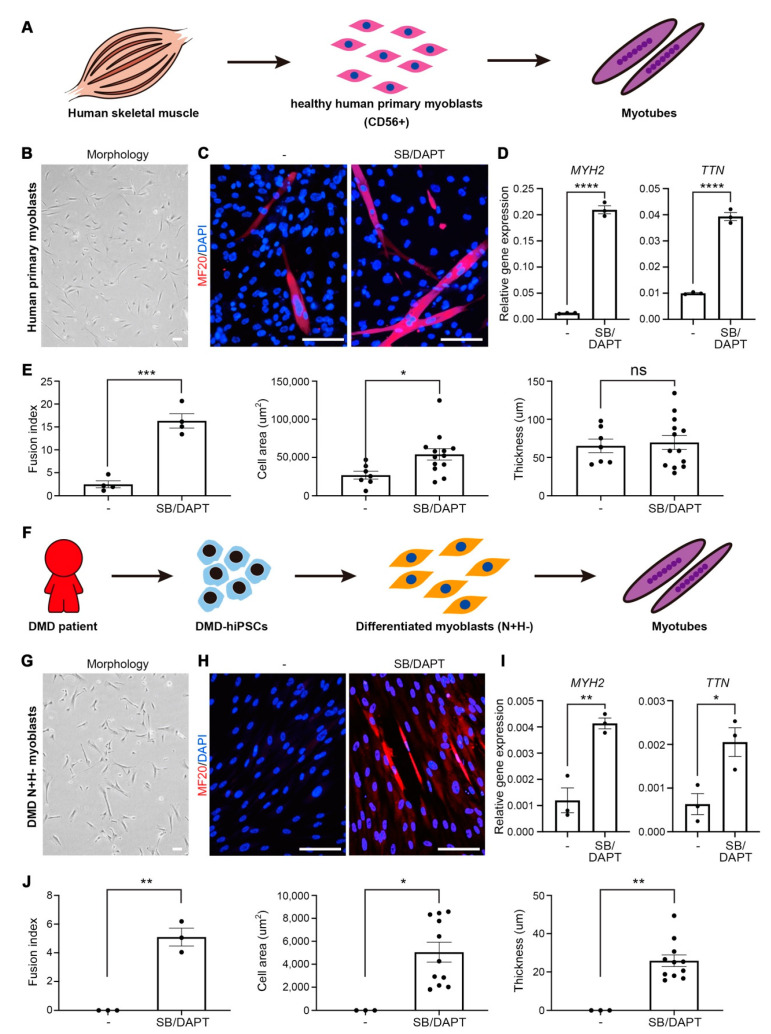
Myotube formation of primary myoblasts from healthy human skeletal muscle and NCAM+/HNK- myoblasts from DMD patient iPSCs with 2 small molecules. (**A**) Schematic illustration of the myotube formation process from human skeletal muscle to in vitro myotube formation. (**B**) A representative image of healthy human primary myoblasts. (**C**) Immunofluorescence image of MF20-stained myotubes by primary myoblasts with or without two small molecules. (**D**) Relative mRNA expression levels of myotube relative marker genes. (**E**) Fusion index, cell area, and thickness of myotube formation from primary myoblasts. (**F**) Schematic illustration of the myotube formation process from DMD patient to in vitro myotube formation. (**G**) A representative image of DMD patient-derived N+H- myoblasts. (**H**) Immunofluorescence image of MF20-stained myotube by DMD myoblasts with or without two small molecules. (**I**) Relative mRNA expression levels of myotube relative marker genes. (**J**) Fusion index, cell area, and thickness of myotube formation from NCAM+HNK1- myoblasts derived from DMD patient iPSCs. Scale bars = 100 um, * *p* < 0.05, ** *p* < 0.01, **** *p* < 0.001, **** *p* < 0.0001, Unpaired *t-test*.

To corroborate the effect of small molecules on promoting myotube formation, we additionally verified that myotube formation was improved by treating these compounds to healthy human primary myoblasts. Primary myoblasts were obtained from human skeletal muscle tissues using the antibody against the surface protein CD56 [12] and were applicable to test for myotube formation by adding selected combinational small molecules (Figure 5a). Healthy human primary myoblasts displaying typical morphological features (Figure 5b) were expanded in the myoblast-maintaining medium, and the effect of combinational treatment of SB-431542 and DAPT during myotube formation was validated using image-based analysis with immunostaining against MF20 protein. Expectedly, the combinational treatment enriched thick and elongated MF20-positive multi-nucleated myotubes (Figure 5c), as well as expression of *MYH2* and *TTN* compare to non-treated myotubes (Figure 5d). Quantifications of fusion index, cell area, and thickness of myotubes clearly showed the enhancement in myotube formation ability by combinational treatment (Figure 5e). Since abnormal upregulation of BMP and TGFβ has been identified in our previous study as pathogenesis leading to myotube defects in Duchene muscular dystrophy (DMD) [9], we next evaluated the combinational treatment as a possible therapeutics for potentiating the ability of DMD patient’s iPSC-derived myoblasts for the myotube formation (Figure 5f). Myoblasts differentiated from the DMD-patient iPSC showed the typical morphology after FACS isolation (Figure 5g) comparable to human primary myoblasts. Importantly, the treatment of selected compounds ameliorated the fusion ability of DMD-myoblasts, confirmed by the presentation of MF20-positive myotubes (Figure 5h) with augmented expression of *MYH2* and *TTN* (Figure 5i). Quantitative evaluation of fusion index, cell area, and thickness after combination treatment showed increases in cell area and thickness of multinucleated cells and myotubes observed with expression of MF20 compared to myotubes of extremely scarcely formed DMD myoblasts (Figure 5j).

## 4. Discussion

The clinical manifestations of myopathic diseases involve loss of muscle cells and poor regeneration, which is attributed to the abnormal regulation of myoblasts, which play an important role in myotube formation during skeletal muscle tissue development and repair of muscle damages [25]. To improve the pathological condition of skeletal muscle tissue, research from innovative perspectives such as cell therapy is being attempted [26], however, countermeasures against the repeated pathophysiological damage that occur continuously in skeletal muscle tissue should be considered. Indeed, the accumulation of myoblasts that fail to produce myofibers is observed in the muscle tissue of patients with muscular dystrophy [27,28,29], and it is difficult to expect therapeutic effects since the persistent pathological conditions also affect the transplanted myoblasts. Therefore, effective cell transplantation therapy requires control that promotes myotube formation of both transplanted and endogenous myoblasts.

To discover drugs that promote myotube formation in myoblasts, either as endogenous or as cell therapeutic agents, we developed an in vitro screening platform using hiPSC-derived myoblasts (Figure 1a and Figure 2a). In our screening, it was found that inhibition of TGFβ or NOTCH signaling pathways encouraged myotube production from myoblasts (Figure 2b), and moreover, combined inhibition of TGFβ and NOTCH more effectively promoted myotube formation (Figure 3a). The role of the TGFβ or NOTCH signaling pathway in the myogenic process has been proposed in a previous study [19], however, the cooperative mechanism between the signaling pathways has not been explained in the myogenic context. Our results show that TGFβ inhibition in myoblasts promotes myotube formation through downregulation of NOTCH-responsive gens (Figure 4h), which is comparable to other studies that TGFβ is involved in the maintenance of undifferentiated myoblasts [30]. Furthermore, the inhibition of NOTCH signaling abolished the effect of TGFβ (Figure 4b,c,g,h), indicating that the myotube generating process is a target regulated by the combinatorial mechanism of TGFβ and NOTCH [22]. To corroborate the effect of the combinational inhibition of TGFβ and NOTCH on the promotion of myotube formation, we employed human primary myoblasts, another model system that could rule out biological or technical errors in studies with iPSCs [31]. The promotion of myotube formation by combinational inhibition of TGFβ and NOTCH was reproduced in the primary myoblasts-based experiment (Figure 5a–c), and furthermore, the effect on myotube formation was also demonstrated in iPSC-derived myoblasts from DMD patients (Figure 5f–j). According to our gene expression database for myogenesis which was previously established [11], the NOTCH pathway is activated in myoblasts and inactivated during myotube production (Figure 4d,e). The promotion of myotube formation by forced inhibition of the NOTCH signaling pathway proposed in this study is a concomitant change in physiological myogenic processes and is therefore expected to stimulate endogenous and transplanted myoblasts.

In addition, the role of TGFβ under the condition of muscle damage is known to stimulate MSC to promote ECM (extracellular matrix) secretion and thereby induce fibrosis [23,32]. However, the role of TGFβ on a single population like myogenic cells other than histological results is still controversial. Our results indicated that stimulation of the TGFβ signaling pathway blocked myotube formation (Figure 2a) and rather promoted proliferation of myoblasts (Figure 4a,b), suggesting that TGFβ prioritizes proliferation of myoblasts over differentiation during the fate selection process in damaged conditions. Moreover, it was considered that stimulation of TGFβ does not convert myoblasts to myofibroblasts as TGFβ-treated myoblasts maintained the ability for myotube formation (Figure 4c).

Although our study confirmed the effect that the cooperative mechanism of TGFβ and NOTCH promotes myotube formation in vitro, it is still unclear whether both inhibitions enhance a positive effect in complex conditions with various cells, such as in vivo conditions. This will have to be addressed in the future by utilizing a screening platform composed of multiple cell types to mimic the in vivo state.

## 5. Conclusions

We report an in vitro screening platform based on hiPSC-derived myoblasts for the discovery of small molecules that promote myotube-forming ability. Small-scale screening using this platform verified that the combinational inhibition of TGFβ and NOTCH promoted myotube formation effectively.

## Figures and Tables

**Table 1:** The list of small molecules for myotube formation.

Small Molecule	Full Name	Concentration
FGFs	FGF2	10 ng/mL
	FGF8	100 ng/mL
CHIR	CHIR99021	3 uM
LY	LY294002	1 uM
PD	PD173074	1 uM
LDN	LDN193189	50 nM
SB	SB431542	10 uM
PMP	Purmorphamine	0.5 uM
XAV	XAV939	2 uM
DAPT	DAPT	10 uM
BMP	BMP4	2 ng/mL
RA	Retinoic Acid	1 uM
TGFs	TGF β1	10 ng/mL
	TGF β2	10 ng/mL
	TGF β3	10 ng/mL
PMA	Phorbol 12-myristate 13-acetate	10 nM

## Data Availability

The data presented in this study are available on request from the corresponding author.
